# STARGATE-X: a Python package for statistical analysis on the REACTOME network

**DOI:** 10.1515/jib-2022-0029

**Published:** 2023-09-21

**Authors:** Andrea Marino, Blerina Sinaimeri, Enrico Tronci, Tiziana Calamoneri

**Affiliations:** Computer Science Department, Sapienza University of Rome, Rome, Italy,; LUISS University, Rome, Italy, http://impresaemanagement.luiss.it

**Keywords:** biochemical reaction networks, network analysis, pathways, Reactome, StARGate-X

## Abstract

Many important aspects of biological knowledge at the molecular level can be represented by *pathways*. Through their analysis, we gain mechanistic insights and interpret lists of interesting genes from experiments (usually omics and functional genomic experiments). As a result, pathways play a central role in the development of bioinformatics methods and tools for computing predictions from known molecular-level mechanisms. Qualitative as well as quantitative knowledge about pathways can be effectively represented through *biochemical networks* linking the *biochemical reactions* and the compounds (*e.g.*, proteins) occurring in the considered pathways. So, repositories providing biochemical networks for known pathways play a central role in bioinformatics and in *systems biology*. Here we focus on Reactome, a free, comprehensive, and widely used repository for biochemical networks and pathways. In this paper, we: (1) introduce a tool StARGate-X (*STatistical Analysis of the* Reactome *multi-GrAph Through* nEtworkX) to carry out an automated analysis of the connectivity properties of Reactome biochemical reaction network and of its biological hierarchy (*i.e.*, cell compartments, namely, the closed parts within the cytosol, usually surrounded by a membrane); the code is freely available at https://github.com/marinoandrea/stargate-x; (2) show the effectiveness of our tool by providing an analysis of the Reactome network, in terms of centrality measures, with respect to in- and out-degree. As an example of usage of StARGate-X, we provide a detailed automated analysis of the Reactome network, in terms of centrality measures. We focus both on the subgraphs induced by single compartments and on the graph whose nodes are the strongly connected components. To the best of our knowledge, this is the first freely available tool that enables automatic analysis of the large biochemical network within Reactome through easy-to-use APIs (*Application Programming Interfaces*).

## 1 Introduction

Bioinformatics holds the promise to decrease the time and cost of the search for predictive biomarkers, therapeutic targets, patient stratification, novel drugs, and therapies. Roughly speaking, those aims are pursued through two main approaches: either artificial intelligence (namely, machine learning) based methods, making predictions from a suitably wide collection of clinical data, or mechanistic methods, making predictions from well-established biological mechanisms (thereby allowing leveraging on the wealth of knowledge available from biology as well as molecular medicine).

Many important aspects of biological knowledge at the molecular level can be represented through *pathways*. As a result, pathways play a central role in the development of bioinformatics methods computing predictions from known well-established molecular-level mechanisms.

Shortly, a *pathway* is a sequence of interactions between molecules that can lead either to the production of a new molecular product (*e.g.,* protein or fat), to the regulation in the expression of a gene, or to a *physical* effect (*e.g.,* cell movement).

Biological pathways play a crucial role in understanding the different processes inside a cell and their analysis is essential for many biomedical studies. For example, many diseases (*e.g.,* cancer, diabetes) stem from the modification/creation/suppression of pathways. The identification of the pathways involved in a specific disease allows the design of personalized strategies for the prevention, diagnosis, and treatment of that disease.

The molecular level experimental knowledge underlying pathways can be represented through *graphs*, namely, *biochemical reaction networks* (*biochemical networks* for short) linking *biochemical reactions* and participating compounds (*e.g.,* proteins). Using biochemical networks, we can represent qualitative as well as quantitative aspects of *pathways* and analyze their interactions (*e.g.,* using SBML simulators like AMICI [1], BioSCRAPE [2], COPASI [3], or libRoadRunner [4]).

A biochemical network provides a mechanistic model for the cell behavior (in fact, a qualitative SBML [5] model can be generated from such a graph) as well as the underlying biological hierarchy (*e.g.,* that of cell compartments). Understanding the properties of such a network, along with those of the underlying biological hierarchy, provides essential insights into the structure of the pathways driving a cell’s behavior.

The above considerations motivate the development of software tools supporting a comprehensive study of the structure of the graph underlying the *biochemical reaction network*.

Comprehensive biochemical networks (encompassing genetic, metabolic as well as signaling pathways) are provided by KEGG [6] and Reactome [7]. Here we focus on Reactome, which is a free, open-source, and open-data knowledge base of bio-molecular pathways. It can be considered as one of the most complete, manually curated, online pathway data sets.

### 1.1 Contributions

We focus on the connectivity properties of Reactome biochemical reaction network as well as of its biological hierarchy (*i.e.*, cell compartments, namely, the closed parts within the cytosol, usually surrounded by a membrane).

Our main contributions can be summarized as follows.

– We propose a tool, StARGate-X (*STatistical Analysis of the* Reactome *multi-GrAph Through* NetworkX) providing a user-friendly Python-based wrapper for the NetworkX.MultiDiGraph class;
– StARGate-X extracts from the Reactome graph a bipartite multi-graph representing reactions and pathways;
– StARGate-X provides both the possibility to exploit the classical NetworkX [8] functionalities to analyze the above generated bipartite multi-graph and *ad hoc* functions for the context of biological pathways;
– Finally, as an example of the use of StARGate-X, we provide an analysis of the Reactome multi-graph, in terms of centrality measures, with respect to in- and out-degree. Namely, we consider the following centrality measures: degree centrality [9], H-index [10], Laplacian [11], leverage [12], and closeness [13] centrality. Detailed definitions of these measures will be given in Subsection 2.1. We focus both on the subgraphs induced by single compartments and on the graph whose nodes are the strongly connected components.

To the best of our knowledge, this is the first freely available tool that enables automatic analysis of the large biochemical network within Reactome through easy-to-use APIs (*Application Programming Interfaces*).

### 1.2 Motivation

Here we propose some possible applications for which StARGate-X capabilities can be used to understand mechanisms of diseases as well as design personalized medical strategies, by providing a (not exhaustive) list of possible applications.

The authors of [14] show the importance of finding central or intermediate nodes that affect the topology of a biological network. Examples of biological questions that can be addressed using graph centrality are: finding molecules in a biological pathway that are not necessarily central but have a crucial biological role in signal transduction or in *Protein-Protein Interaction* (PPI) networks; detecting nodes that interact with many other proteins; finding molecules that are crucial for stimulating the expression of genes.

Using the PPI network across the whole cell (as StARGate-X does through the Reactome network) enables sub-cellular localization prediction of protein-related data (*e.g.*, as in the CELLmicrocosmos PathwayIntegration (CmPI) from [15]). This, in turn, enables the construction of a disease-related protein-protein interaction network. For example [15], shows how this approach can be used to (semi-automatically) construct a MUPP1/MPDZ-related (a major player in the context of dilated cardiomyopathy) interaction network.

In [16], it is shown how intra/inter-compartmental PPI can be used to estimate the efficiency of biological pathways. This, in turn, can be used to support personalized medicine (*e.g.*, [17]) and drug discovery (e.g., [18]).

The work [19] shows how network-based ranking (*i.e.*, centrality analysis) of biological components has been widely used to find influential nodes in large networks, with applications in biomarker discovery, drug design, and drug repurposing.

Azimzadeh et al. [20] presents network-based analyses providing biomarkers (namely, upregulation of the arachidonic acid through the sphingolipid signaling pathway) for *Chronic obstructive pulmonary disease* (COPD). Furthermore, by integrating network-based analyses and clinical data, it shows a strong association between hypoxia and the upregulation of sphingolipids in smokers with emphysema, vascular disease, hypertension, and in those with an increased risk of lung cancer.

Gene network connectivity is highly informative for disease architectures, including heritability [21].

Through an analysis of differentially connected genes, the authors of [22] show that the loss of connectivity is a common topological trait of cancer networks and unveils novel candidate cancer genes. This approach, integrating differential expression, together with the differential connectivity, improves the classic enrichment pathway analysis by providing novel insights into putative cancer gene biosystems.

The work in [23] developed a *Juvenile Idiopathic Arthritis* (JIA) interactome of 2479 proteins from 348 JIA-associated genes. The analysis of such a network revealed that the genes of greatest potential functional importance are PTPN2 and STAT1 (for oligoarticular JIA) and KSR1 (for RF-ve polyarticular JIA). Furthermore, the work identified clusters of 23 and 14 related proteins for oligoarticular and RF-ve polyarticular JIA, respectively.

In [24] an a network-based molecular framework for predicting potential drug targets for rabies infection is presented.

Finally [25], shows how network-based techniques can help in the identification of single-target and multi-target drug candidates. Successful network-based drug development strategies are shown through the examples of infections, cancer, metabolic diseases, neurodegenerative diseases, and aging. The same work also provides examples of network-based drug repurposing.

### 1.3 Related work

Broadly speaking, the most common types of biological pathways are: metabolic, genetic, and signal pathways. Thanks to the progress in high-throughput technologies, there has been an expanding knowledge about pathways. Thus, in the last 15 years, academics and commercial groups have created and maintained a number of pathway databases, for example KEGG [6], Reactome [7], WikiPathways [26], Pathway Commons [27], Pathway Interaction Database (PID) [28]. Rationales for the construction of such databases are outlined *e.g.,* in [29]. Topology-based methods for their analysis can be found *e.g.,* in [30]. Finally, the impact of database selection on *-omics* analysis has been studied in [31].

The above databases differ in many aspects. For example, some of them focus on specific organisms (*e.g.,* EcoCyc [32]), a few others focus on a particular disease or disorder (*e.g.,* The Cancer Cell Map [33]), some contain only metabolic pathways or only signaling pathways and some contain both (*e.g.,* KEGG [6], Reactome [7]). Among the latter, Reactome is free, open-source, and open-data. From this, it stems our focus on it.

As most of the biological pathway databases, Reactome stores its content in a relational database. The same data are also saved in a graph database [34] implemented through Neo4j [35].

Graph algorithms have been widely used to model and study biological properties (*e.g.,* see [14, 36], [37], [38], [39]). In such a context, the paper closest to ours is [39], which studies the connectivity of the Reactome graph. However [39], only focuses on signaling pathways and, accordingly, disregards simple molecules, such as water and ATP. Furthermore [39], completely ignores the biological hierarchy induced by cell compartments. Our proposed tool (StARGate-X) and our results instead encompass both signaling and metabolic pathways, take into account simple molecules, and do consider the biological hierarchy induced by cell compartments.

Summing up, although graphs have been widely used to model and study biological properties, to the best of our knowledge, no published paper has addressed both signaling and metabolic pathways, has taken into account simple molecules, and has catered for the biological hierarchy induced by cell compartments.

## 2 Preliminaries

A *(directed) graph* *G* = (*V*, *E*) is a data structure constituted by a set *V* of entities called *nodes* and a set *E* ⊆ *V* × *V* of ordered pairs called *directed edges* (or, wherever no confusion arises, simply *edges*) defining binary relations. If order does not matter in the binary relation between nodes *u* and *v*, then both (*u*, *v*) and (*v*, *u*) are in *E* and the edge is *undirected*.

For any node *v* of *G*, its *in-neighborood* is set *N* ^−^(*v*) = {*u* ∈ *V* (*u*, *v*) ∈ *E*} and its *out-neighborood* is set *N* ^+^(*v*) = {*u* ∈ *V* (*v*, *u*) ∈ *E*}; set *N*(*v*) = *N* ^+^(*v*) ∪ *N* ^−^(*v*) is called *neighborhood* of *v*. The *in-degree* (respectively *out-degree*) of *v* is the cardinality of set *N* ^−^(*v*) (respectively *N* ^+^(*v*)), that is the number of edges incoming in (respectively outcoming from) *v*; the *degree* of *v* is the sum of in- and out-degrees.

A *path* in *G* is a sequence of nodes *v*_1_, *v*_2_, … *v*_*k*_ such that *v*_*i*_ ≠ *v*_*j*_ for every *i*, *j* = 1, …, *k*, *i* ≠ *j*, and (*v*_*i*_, *v*_*i*+1_) is in *E* for every *i* = 1, … *k* − 1; it is a *cycle* if edge (*v*_*k*_, *v*_1_) ∈ *E*.

A graph is *strongly connected* if there exists a path between every ordered pair of nodes. If *G* is not strongly connected, it can be partitioned into its maximal strongly connected components, *i.e.* in the node equivalence classes with respect to the relation of being linked by a directed path.

A *directed acyclic graph (DAG)* is a graph without any cycle. *G* is a DAG if and only if it has no strongly connected subgraphs with more than one node.

If each strongly connected component of a graph *G* is contracted to a single node, the resulting graph is a DAG, called *condensation of* *G*, that we will denote by *G*_*SCC*_.

A *weakly connected component* of a directed graph is a subgraph whose nodes are connected by an undirected path when the direction of edges is ignored.

### 2.1 Centrality measures

Our implementation includes all the functionalities offered by NetworkX [40] for graph analysis. In particular, we consider different graph centrality measures. Graph centrality measures are widely used in systems biology to identify influential nodes within a biological network. For example, in [41], it has been shown that nodes with high degrees (*i.e.* nodes that have many links with the rest of the graph) in protein interaction networks are often functionally important, and the deletion of such nodes can be related to lethality. A recent survey on the use of centrality measures to classify nodes in protein-protein interaction networks can be found in [19]. Along the same lines, centrality measures are used to identify influential nodes in biochemical reaction networks. For example [42], uses centrality to identify important nodes in signaling networks for breast cancer cell lines. Here we discuss and compare different centrality measures (*i.e.*, degree centrality, H-index, Laplacian, leverage, and closeness centrality). Some of them are already used in analyzing biological networks, while others are transferred from different fields of science, such as social network analysis.

As the graph we extract represents a biochemical reaction network, in- and out-degree have different meanings according to the considered nodes. Namely, the in-degree of a reaction represents the number of its reactants while the out-degree is the number of its products; the in-degree of a physical entity represents the number of reactions producing it while its out-degree is the number of reactions it can be involved in.

Based on the concepts of degree, in- and out-degrees, we divide centrality measures into: (i) *out-centrality measures*, which indicate a version of the centrality measure that can capture the importance of a node as a sender of information, (ii) *in-centrality measures*, that can capture the importance of a node as a receiver of information, (iii) *total-centrality measures* that can be seen as a combination of (i) and (ii) and which produce a centrality score for each node in a network that quantifies the importance of each node both upstream and downstream.

Moreover, we distinguish between local and global centrality measures: the first ones are based on local information (concerning either single nodes or, at most, their neighbors), while the second ones capture information carried out by the whole data structure.

Here we considered several centrality measures already used in biological network analysis (see, for example, [43]) while we excluded others, such as betweenness and coreness centralities, because they do not apply to multi-graphs.

In order to make this paper self-contained, we recall the definitions of the measures considered.

– The *degree centrality* (*DC*) is a local measure that assigns importance to a node only based on its degree. In directed graphs, we can define two degree centralities, depending on whether we consider only in- or out-degree. Formally, *DC* ^+^(*v*) = *d* ^+^(*v*) and *DC* ^−^(*v*) = *d* ^−^(*v*). The degree centrality was first defined in [9] and has been used successfully in biological network analysis, *e.g.*, [41].
– The *H-index* (*HC*) is a local measure originally introduced teHirsch2005 to measure the citation impact of a journal. Compared to degree centrality, the *H*-index is usually considered a better indicator of a node’s influence on spreading dynamics (see *e.g.* [44]). The *H*-index of a node *v* is defined as the maximum value *h* such that there exist at least *h* in- (out-) neighbors of *v* with a degree no less than *h*. Formally, if we denote by $N_{\leq h}^{+}\left(v\right)$ the neighbors of *v* that have at least degree *h*, we have $HC^{+}\left(v\right)=\max_{1\leq h\leq d^{+}\left(v\right)}\min\left(|N_{\leq h}^{+}\left(v\right)|,h\right)$ . *HC* ^−^(*v*) is defined similarly.
– The *Laplacian centrality* (*LAPC*) was originally introduced in [11] with the objective to reveal more structural information about the connectivity of the subgraph around a node *v* and thus further than its immediate neighborhood as considered by degree centrality and *H*-index. Hence, it can be considered as an intermediate between global and local centrality measures of a vertex. Intuitively, the removal of nodes with high *LAPC* would significantly impact the network. Formally, $LAPC^{+}\left(v\right)={\left(d^{+}\left(v\right)\right)}^{2}+d^{+}\left(v\right)+2\sum_{u\in N^{+}\left(v\right)}d^{+}\left(u\right)$ . *LAPC* ^−^(*v*) is defined similarly.
– The *leverage centrality* (*LC*) is a local measure introduced [12] to capture the relationship between the degree of a given node and the degree of each of its in- (out-) neighbors, averaged over all in- (out-) neighbors. Intuitively, a node with high *LC* has a higher degree than its neighbors. Formally:
$$LC^{+}\left(v\right)=\frac{1}{d^{+}\left(v\right)}\underset{u\in N^{+}\left(v\right)}{\sum }\frac{d^{+}\left(v\right)-d^{+}\left(u\right)}{d^{+}\left(v\right)+d^{+}\left(u\right)}.$$
  *LC* ^−^(*v*) is defined similarly.
– The *closeness centrality* (*CC*) is a global measure originally introduced in [13] and measures the average inverse distance of a node to all the others. Nodes with a high closeness score have the shortest distances to all other nodes. It is considered a way of detecting nodes that are able to spread information very efficiently because it measures the influence of a node by computing the number of the shortest paths in the whole graph, so it is, in general, not suitable for large graphs due to its high computational complexity. Formally, if we denote by *n* = *V*(*G*) the number of all the vertices of the graph and by *d*_*G*_(*u*, *v*) the length of a shortest path between nodes *u* and *v* in *G*, we have $CC\left(v\right)=n/\left(\sum_{u\in V}d_{G}\left(u,v\right)\right)$ .

## 3 Description of STARGATE-X

We propose a Python implementation that provides a graph representation of the Reactome database. In the following, we refer to this implementation as StARGate-X, and its code is available at https://github.com/marinoandrea/stargate-x.

When choosing a graph model for a biological network, one needs to identify which biological entities are associated with nodes and what is the meaning of the connections between them. In this context, depending on the question to tackle, different types of graphs have been proposed in the literature, for example: bipartite graphs, directed graphs, hypergraphs, etc. (*e.g.* see [28]).

Reactome contains many entities, each one decorated with a number of additional information. Namely, there are physical entities (for which the cell compartments where they lie are indicated, and notice that two occurrences of the same component lying in different compartments are considered as two different components), reactions (having some components as input and some other components as output), catalysts (substances, *e.g.* some enzymes, that speed up a reaction but are not consumed or altered in the process), regulators (genes involved in controlling the expression of one or more other genes, they can be either positive or negative) and black boxes (not referable to any exact reaction but used as a placeholder of experimentally known mechanisms, although not formally defined).

In this paper, we extract from Reactome a bipartite graph. Namely, we define two kinds of nodes, those representing *physical entities* and those representing *events* (*e.g.* reactions). The directed edges, connecting the nodes, are partitioned into three types: *input* edges (from components to events they are involved in), *output* edges (from events to their component products), and *modifiers* (from components to events they influence, these edges are partitioned into positive or negative regulators and catalysts).

Notice that our data structure is a *bipartite multi-graph*, which seems to us as the most suitable structure to represent pathways: it is more flexible than a bipartite graph but sufficiently simple to allow for computationally efficient analysis.

We propose a Python implementation that provides a wrapper for the networkx.MultiDiGraph class. Each graph instance is associated with a certain species and can be either pre-built and loaded or directly built from a Neo4j graph database instance running on the user’s machine. This latter option is preferred as it allows to control the version of the Reactome’s database to work with. Once the graph is built, the tool provides some classical functionalities to analyze it. Namely, on the one hand, it is possible to exploit all functionalities of NetworkX in order to extract information from the graph. On the other hand, it provides *ad hoc* functions for the pathway context, such as the generation of special subgraphs starting from a given pathway or a compartment.

Notice that, although the Neo4j database can be also used for graph analysis, the Python implementation offers different advantages. Indeed, the Python package is built on NetworkX, which is designed more for research and thus offers the possibility to implement complex algorithms in an easier and more efficient way. Moreover, although Neo4j offers a nice interactive browser visualization, NetworkX has more options for displaying graphs allowing us to use different visualization algorithms depending on the application we are interested in.

We conclude this section observing that StARGate-X complements the Neo4j implementation of Reactome (one of the largest pathway public repositories), in particular by making it easier to implement new complicated algorithms or aggregate functions to exploit the graph structure of Reactome. It would be more cumbersome to do this directly in Neo4j. Finally, we note that to use our tool with a different pathway repository, it would be necessary to store Pathway data in the graph database according to Reactome ’s schema for labels and nodes. As discussed in 6, in future versions of StARGate-X, we plan to include functionalities that would allow to import data from different repositories and expose it in a standardized way to the user.

### 3.1 Information extracted from REACTOME and methodology

The development of StARGate-X was carried out in several steps. At first, the open source graph database containing pathway information provided by the Reactome team (available at https://reactome.org/download-data) was downloaded. This database includes pathways for 84 different species. Once downloaded, we set up a local Neo4j instance for accessing the data. Then, after the relevant entities and relationships were identified (see Section 3), a set of Cypher queries were produced in order to extract them for each species directly from the Neo4j instance. Such queries target the following Reactome elements:

– All reaction nodes (*i.e.*, ReactionLikeEvent) for which the relationship species targets the species of interest or whose identifier matches the regex R-(ALL—NUL)-.* (which means the reaction is generic and does not pertain to a single species).
– All compounds (*i.e.*, PhysicalEntity) that are involved in the following Reactome relationships: input, output, referenceEntity, and regulator. As a postprocessing step for regulators, we differentiate between compounds that are bound to PositiveRegulation nodes from NegativeRegulation nodes.
– All compounds (*i.e.*, PhysicalEntity) that are involved in the physicalEntity relationship where the target participates in the catalystActivity relationship.

These queries are used within the ReactomGraph.build function in the StARGate-X package, which takes an identifier for the selected species (and optional Neo4j client configuration options), and returns an instance of a networkx.MultiDiGraph.

## 4 Example usage of STARGATE-X

As our tool offers a Python interface to operate on a specific graph representation of the database, its use is not limited to the analysis we discuss in Section 5. By extending one of NetworkX’s base classes, StARGate-X allows for any kind of graph-based data exploration and/or simulation on the graph using a widely adopted interface. In the following, we provide three possible example usages.

In Listing 1, we show an example where we analyze the connectivity features of a single node in a specific compartment and for a certain pathway. Here both StARGate-X and NetworkX specific functionalities are used.
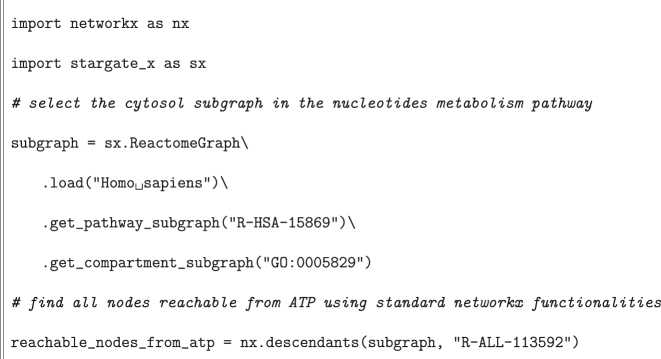

**Listing 1**

Example with connectivity.

Listing 2 shows how we can obtain some centrality measures for all nodes in a specific pathway. As these measures are not available in NetworkX out of the box, we introduced them in the package while conducting our analysis.
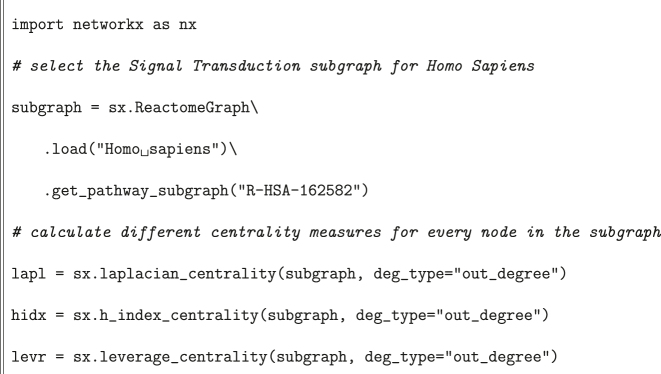

**Listing 2**

Example with centrality measures.

Finally, in Listing 3, there is an example of how to obtain participating compounds given a reaction node. Specifically, we find all compounds participating in the Phosphorylation of complexed TSC2 by PKB within the Signal Transduction pathway.
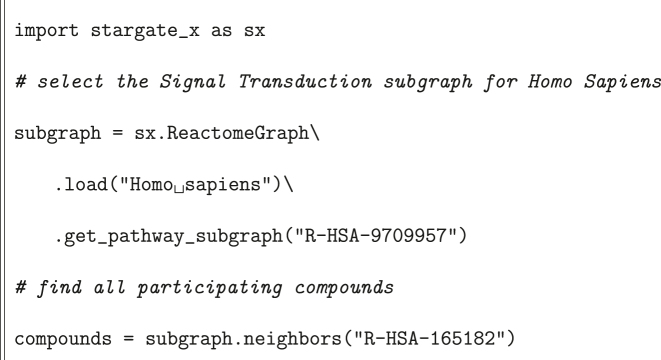

**Listing 3**

Example with reaction compounds.

## 5 Analysis of the REACTOME multi-graph through STARGATE-X

To investigate complex and large biological networks, different analysis methods have been developed from other fields. In particular, one approach is to analyze their topological structure and try to relate this to biological functions. We performed such analysis on the multi-graph constructed through our tool; in this section, we detail the results of our experiments. As a proof-of-concept to illustrate the utility of our tool, we focus only on the human biochemical reaction network, and from now on, we refer by *G*_*R*_ to the multi-graph extracted from the Reactome database.

### 5.1 Experimental setting

All the experiments of this section were carried out on an AMD Ryzen 3700x CPU (@3.6 Ghz, 8 cores/16 threads), 16 GB of DDR4 RAM clocked at 3200 Mhz, and 500 GB of SSD storage (NVMe 1.3, PCIe 3.1). We ran our experiments on Linux (distribution Ubuntu 20.04 running in WSL 2.0) using Python version 3.8.5. Our dockerized Neo4j instance (v3.5) hosts version 73 of Reactome’s graph database.

### 5.2 Graph *G*_*R*_ and its condensation

We focus on the set of pathways related to *H. sapiens*. Using StARGate-X, we compute the multi-graph *G*_*R*_, which has 34,931 nodes and 52,654 arcs. Among its nodes, 13,148, *i.e.* 37.6%, are event nodes (*e.g.*, reactions), while the remaining 21,783, *i.e.* 62.3%, represent physical entities (*e.g.*, biochemical molecules). For what concerns *G*_*R*_ edges, 24,937 of them, *i.e.* 47.3%, are input relations (linking a reactant to a reaction), 19,959, *i.e.* 37.9%, are output relations (linking a reaction to one of its products), 5703, *i.e.* 10.8% catalysts (linking a catalyst molecule to a reaction), 1393, *i.e.* 2.6%, positive regulations and 662, *i.e.* 1.2%, negative regulations.

Starting from our multi-graph *G*_*R*_, we constructed the condensation *G*_*SCC*_ (whose nodes are the strongly connected components). It turns out that this DAG has 22,795 nodes and 25,773 arcs. Moreover, 9019 nodes are sources and involve 106 compartments out of 111. There exists a giant strong connected component including about 32.4% of the nodes of the original graph and it includes nodes from 77 out of 111 compartments; about 65% of the nodes constitute a single node component and the remaining 3% are not trivial graphs with few tens of nodes including nodes from at most 3 compartments. The diameter of this DAG is 34.

Moreover, *G*_*SCC*_ has 655 weakly connected components. Among them, only one is huge and includes 19,639 nodes (that are strongly connected components of *G*), while the remaining ones have less than 50 nodes (and most of them have less than 5 nodes). If we sort these weakly connected components according to their increasing number of nodes in the original graph database, the about 40 first ones include single chemical events associated with diseases. These components include nodes from at most 6 different compartments.

### 5.3 Centrality measures

We first study the average in- and out-degree centrality of the nodes, where the average is computed on nodes of the same category.

**Figure 1:**
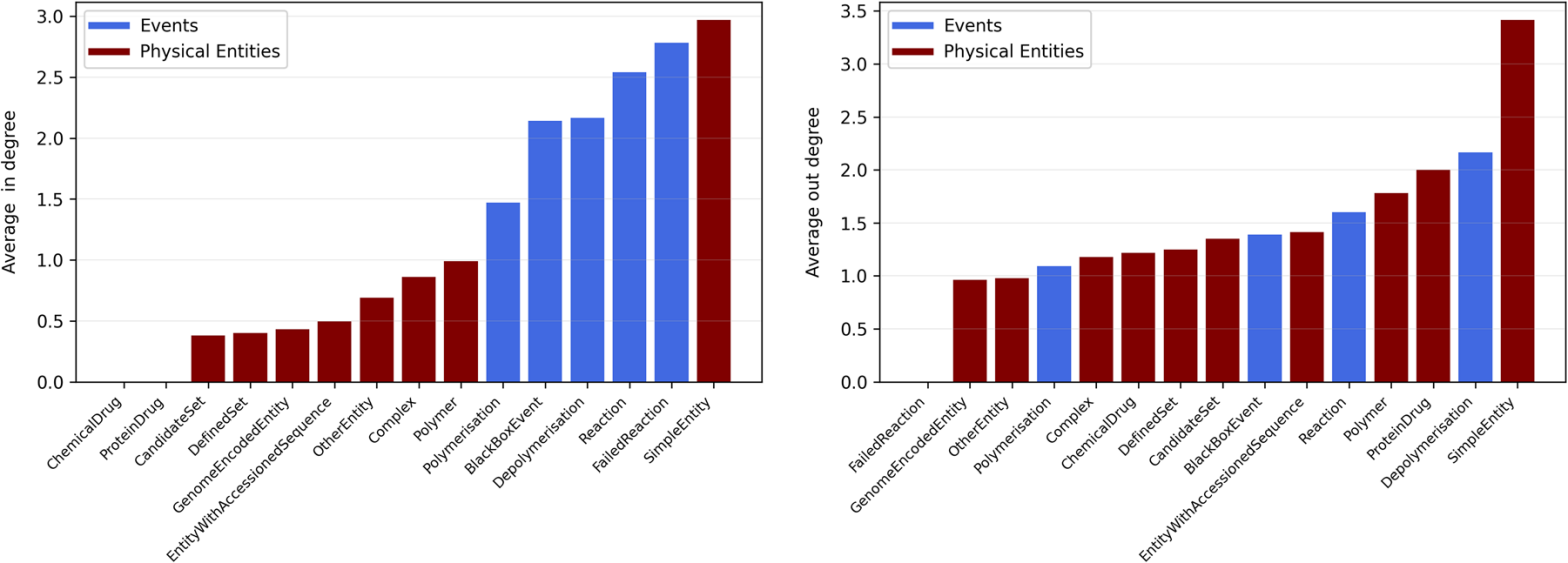
Average in- and out-degree for each Reactome node category.

Interestingly, the results (see Figure 1) show that, while simple entities have maximum both in- and out-degree, in general, the sorting w.r.t. the in- and out-degree is far from being the same, as especially highlighted by the rectangles representing the events.

This motivates the following study of each one of the centrality measures.

For each of the centrality measures defined in Section 2, we show the following statistical information: (i) the cumulative distribution function (CDF) *F*(*x*), *i.e.* the percentage of network nodes whose feature values is less than or equal to *x* and, (ii) the probability density function (PDF), *i.e.* the probability that the feature takes value *x*.

**Figure 2:**
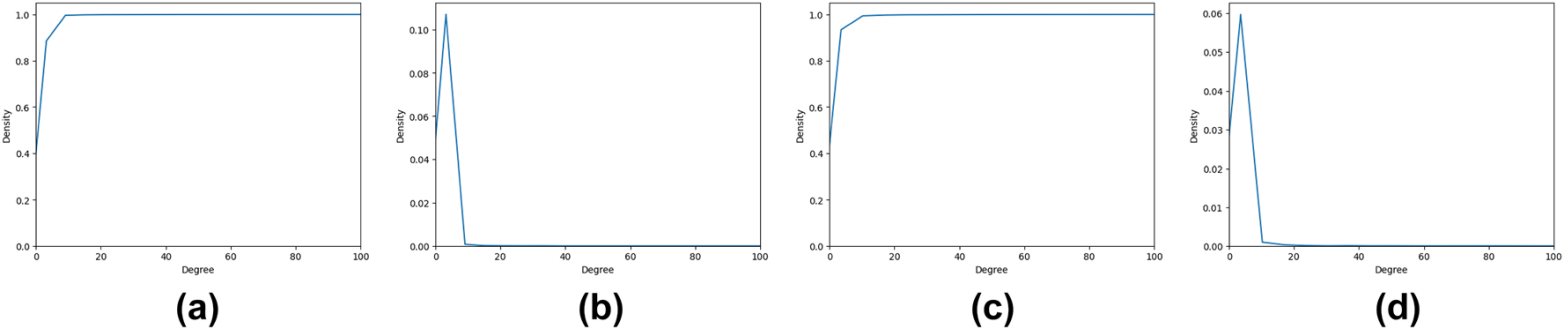
The CDF and PDF for *DC* measure. (a) *DC* ^−^ measure CDF. (b) *DC* ^−^ measure PDF. (c) *DC* ^+^ measure CDF. (d) *DC* ^+^ measure PDF.

Figure 2(a)–(d) show respectively, CDF and PDF, for the in- and out-degree centrality measure.

**Figure 3:**
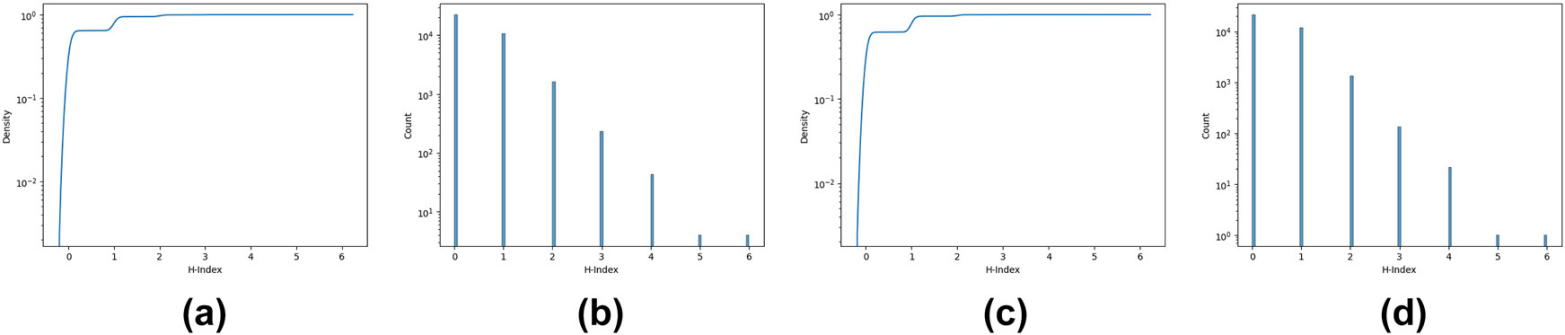
The CDF and PDF for *HC* measure. (a) *HC* ^−^ measure CDF. (b) *HC* ^+^ measure vs N. of nodes. (c) *HC* ^+^ measure CDF. (d) *HC* ^+^ measure vs N. of nodes.

As for the H-index (through the in- and out-degrees) of network nodes, Figure 3(a) and (c) show the CDF for this measure. As the H-index assumes only few values in this dataset, we choose to present the histogram of the frequency of these values instead of the PDF function in Figure 3(b) and (d).

**Figure 4:**
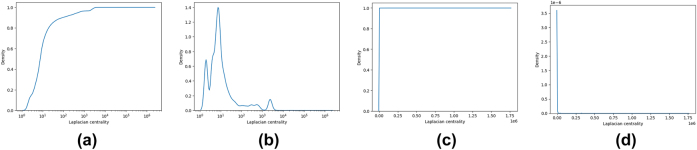
Laplacian centrality. (a) *LAPC* ^−^ measure CDF. (b) *LAPC* ^−^ measure PDF. (c) *LAPC* ^+^ measure CDF. (d) *LAPC* ^+^ measure PDF.

For what concerns the Laplacian measure, Figure 4(a)–(d) show respectively, CDF and PDF for in- and out-degrees.

**Figure 5:**
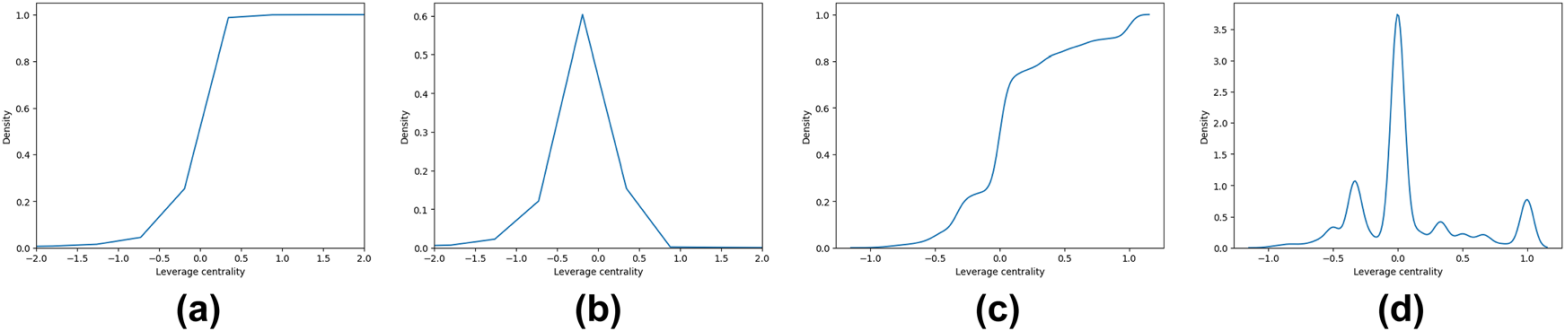
The CDF and PDF for the *LC* measure. (a) *LC* ^−^ measure CDF. (b) *LC* ^−^ measure PDF. (c) *LC* ^+^ measure CDF. (d) *LC* ^+^ measure PDF.

As for the leverage (through the in-degree) of network nodes, Figure 5(a) and (b), show respectively, CDF and PDF for in- and out-degrees.

**Figure 6:**
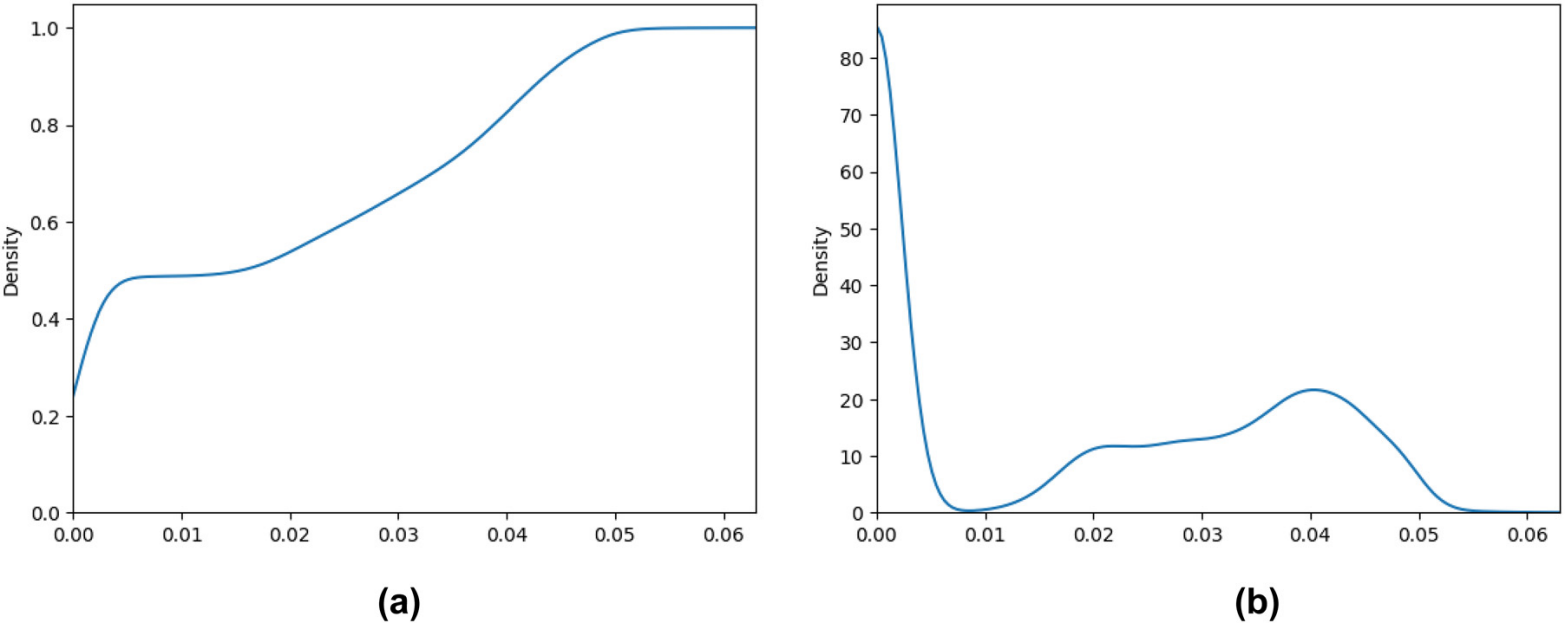
The CDF and PDF for the *CC* measure. (a) *CC* measure CDF. (b) *CC* measure PDF.

Finally, Figure 6(a) and (b), show respectively, CDF and PDF for the total degree of the closeness centrality measure.

We can observe that the distribution of the degree centralities in Figure 2(b) and (d) forms a power low which is usually expected for social networks. Only 104 (206) nodes have an in-degree (out-degree) larger than 10 (*i.e.* less than 0.3% (0.59%)) of the nodes. Clearly, a node with a high in-degree does not necessarily have also high out-degree. For example, ATP and ADP are the ones involved in reactions to store and release energy. Thus, a node corresponding to ADP in the cytosol (ref. *R* – *ALL* – 29,370 in Reactome) has the largest in-degree, equal to 1163, (as it is the output of many reactions). Similarly, a node corresponding to ATP in the cytosol (ref. *R* – *ALL* – 113,592 in Reactome) has the largest out-degree, equal to 1324.

A similar argument can be made for the H-index and Laplacian measure, where we can see that there are few nodes with high values and most of the nodes have a value near 0.

Moreover, for these measures DC and HC, the graphics for the out- and in-measures are almost the same whereas the LAPC, and LC measures represent very different behavior.

### 5.4 Execution time

Our implementation is quite fast in practice. It took us 0.48 and 0.22 s to compute the in- and out-degree for all the nodes. The *HC* was determined in 0.69 s and 3.04 s for the in- and out-degrees, respectively. Finally, although *CC* is theoretically computationally expensive, it has been computed in 286.66 s (less than 5 min) in our graph, thanks to the sparseness of the graph.

### 5.5 Compartment subgraphs

Reactome refers to the Gene Ontology in order to define cell compartments.1
http://geneontology.org/docs/ontology-relations/
 We exploit the relation ‘component of’ in order to produce a hierarchy of compartments. Nevertheless, the result is not a (directed) forest but a DAG; indeed some compartments are components of more than one super-compartment. In fact, Reactome labels components and reactions with one, and more rarely, several compartments that can be either disjoint or laying at a different abstraction level (in some cases, one could contain another one).

Moreover, the compartments may have dimensions even very different, so studying the same measures on the subgraphs induced by all the nodes of every single compartment could be meaningless. For this reason, we produce a subset of more informative subgraphs obtained exploiting the higher-level compartments in the hierarchy.

More in detail, we consider the compartment hierarchy tree from the Gene Ontology, then we add a dummy root and its two children, one related to the cell and the other one to the extracellular region. We discard all compartments without reaction nodes in Reactome and its children are linked to the lowest not discarded ancestor.

In this way, we get 111 compartments, and for each of them, we consider the graph induced by the set of all nodes referred to it.

While the centrality measures are computed globally on the whole network, in this section we focus on the local analysis of the network based on single compartments. For each of them, we consider the following features: number of strongly connected components, number of weakly connected components, and number of nodes.

Note that knowledge of SCCs is useful as they provide an effective method to address one of the main challenges in analyzing biochemical networks: finding attractors (*e.g.*, see [45] and citations thereof).

In the following graphics, we first consider all the compartments (computed after our label adjustment), then we detail the results of only the 10 compartments with the largest number of nodes (see Table 1), in order to increase the legibility of the figures.

**Table 1:** The number of connected components for the top 10 compartments (for which the number of nodes is highlighted).

Compartment	No. SCC	No. WCC	No. nodes
Cytosol	7873	1115	11,728
Plasma membrane	8066	1327	9074
Nucleoplasm	6281	184	8085
Extracellular region	3657	1172	3789
Endoplasmic reticulum membrane	1273	366	1399
Mitochondrial matrix	685	43	1160
Endoplasmic reticulum lumen	644	147	760
Golgi membrane	618	169	647
Golgi lumen	399	47	417
Mitochondrial inner membrane	272	80	374

**Figure 7:**
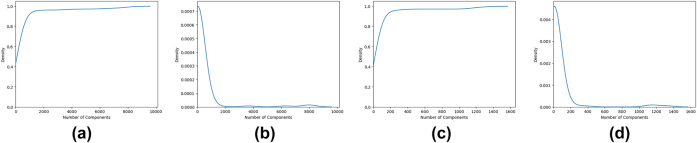
Strongly and weakly connected components number across all the compartments. (a) SCC CDF. (b) SCC PDF. (c) WCC CDF. (d) WCC PDF.

**Figure 8:**
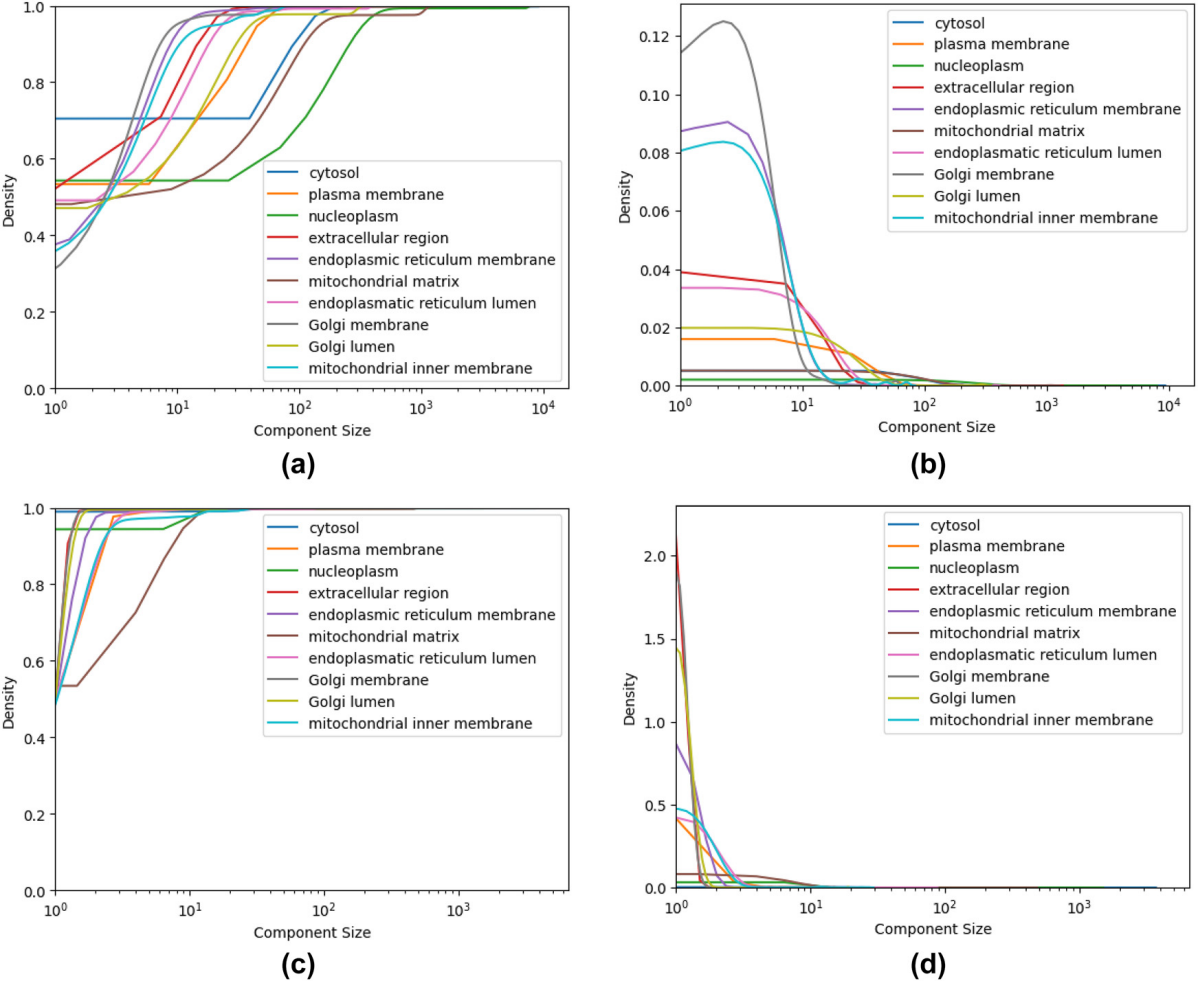
Strongly and weakly connected components size across top ten compartments. (a) WCC CDF. (b) WCC PDF. (c) SCC CDF. (d) SCC PDF.

Figure 7 shows CDF and PDF for the number of strongly and weakly connected components of all compartments, while Figure 8 highlights the behavior of the top ten compartments.

**Figure 9:**
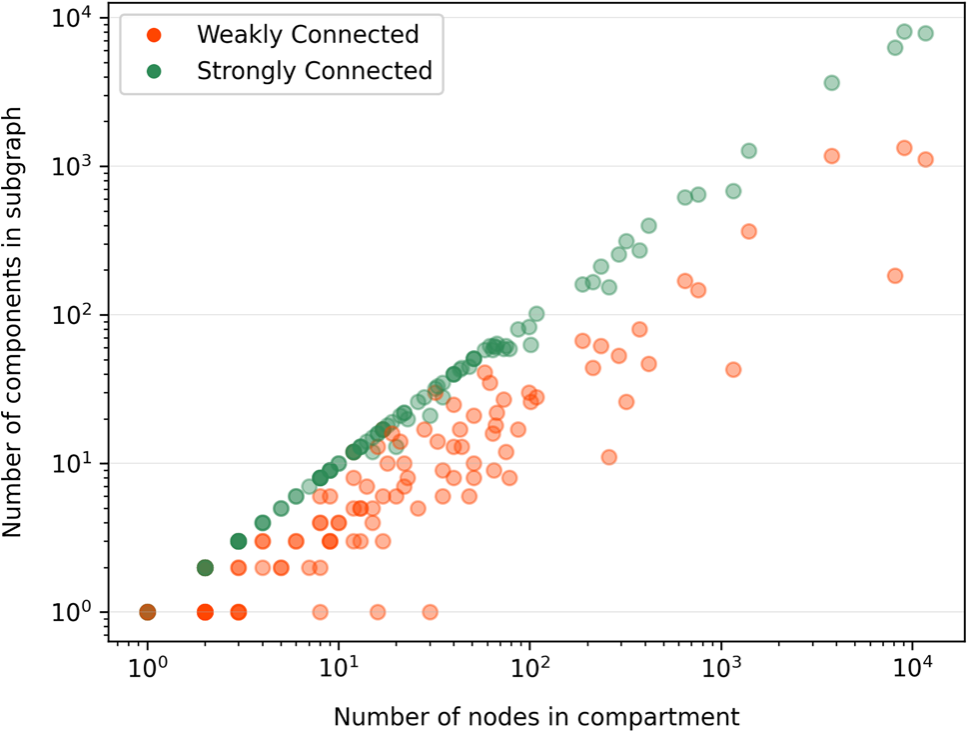
The number of (either weakly or strongly) connected components for each subgraph induced from a cellular compartment by the number of its nodes.

In Figure 9, we show the number of (either strongly or weakly) connected components: on the *x*-axis we represent the compartment subgraphs according to their number of nodes, while on the *y*-axis the number of components is represented. It is worth noting that the number of strongly connected components grows up linearly almost exactly with the number of nodes in the subgraph; vice-versa, the number of weakly connected components, that trivially is smaller than or equal to the number of the strongly connected components, varies in a rather wide range.

## 6 Conclusions

In this paper, we introduced a new tool, StARGate-X, providing a user-friendly Python-based wrapper for the NetworkX.MultiDiGraph class. As an example of the use of StARGate-X, we provide an analysis of the Reactome multi-graph in terms of centrality measures. StARGate-X complements the Neo4j implementation of Reactome (one of the largest pathway public repositories), in particular by making it easier to implement new, possibly complex, algorithms for the analysis of the graph structure of the biochemical network modeled within Reactome. Indeed, it would be much more cumbersome to define such algorithms directly through Neo4j.

In the next future, we aim to extend our tool in order to make it usable for other pathway repositories or even directly on SBML models.
